# What is left after an error? Towards a comprehensive account of goal-based binding and retrieval

**DOI:** 10.3758/s13414-022-02609-w

**Published:** 2022-11-30

**Authors:** Anna Foerster, Birte Moeller, Christian Frings, Roland Pfister

**Affiliations:** 1 Department of Psychology III, University of Würzburg, Würzburg, Germany; 2 Department of Cognitive Psychology, University of Trier, Trier, Germany

**Keywords:** Error processing, Episodic binding, Action control

## Abstract

The cognitive system readily detects and corrects erroneous actions by establishing episodic bindings between representations of the acted upon stimuli and the intended correct response. If these stimuli are encountered again, they trigger the retrieval of the correct response. Thus, binding and retrieval efficiently pave the way for future success. The current study set out to define the role of the erroneous response itself and explicit feedback for the error during these processes of goal-based binding and retrieval. Two experiments showed robust and similar binding and retrieval effects with and without feedback and pointed towards sustained activation of the unbound, erroneous response. The third experiment confirmed that the erroneous response is more readily available than a neutral alternative. Together, the results demonstrate that episodic binding biases future actions toward success, guided primarily through internal feedback processes, while the erroneous response still leaves detectable traces in human action control.

## Introduction

Episodic binding and retrieval shape human action control (Frings et al., 2020; Henson et al., 2014; Hommel, 2004). For one, action episodes are represented in an integrative manner through bindings between features of the action and the acted upon stimuli. Second, encountering these stimuli again retrieves any bound actions, facilitating their selection and execution. Recent studies demonstrated that even if actions go awry, binding and retrieval processes operate swiftly as a corrective force (Foerster, et al., 2022a; Foerster et al., 2021; Parmar et al., 2022). That is, if agents commit an error, the correct response is still bound to relevant and irrelevant stimuli. Thus, binding does not only relate to the events that actually happened but binding also relates to what agents intended to do. Still less clear is whether (a lack of) error awareness plays a role for these binding effects. Here, we ensured error awareness by introducing explicit feedback about the error and analyzed its effect on binding and retrieval processes between the stimuli and the correct response (Experiments 1 and 2). These experiments further suggested that the erroneous action remains in an activated yet unbound state. In a final experiment we therefore elucidated the fate of the erroneous response, probing whether its representation continues to shape action control either alone or through bindings with the correct response (Experiment 3). Before turning to these open issues, however, we will review the current state of the art in research on binding and retrieval in the following.

### Binding and retrieval in perception and action

Modern theoretical accounts of feature binding and retrieval emerged from a series of sophisticated experiments on the structural properties of human perception and action. Pivotal for these developments were neurophysiological insights that showed the human brain to code different features of stimuli in separate cerebral areas, so that, for example, colour, shape, and location of a visual stimulus would give rise to distributed neural activity (Milner, 1974). Forging an integrated percept from these distributed features requires a mechanism that binds all these features together (Singer, 1994; Singer & Gray, 1995; Treisman, 1993, 1996).

Feature binding does not only mediate a single perceptual or action episode, however, but can also affect perception and action whenever previous features are reencountered. Research on feature repetition effects, for example, has suggested that repeating any one of the bound features can retrieve the entire set of features. This observation has first been reported in experimental paradigms on object perception so that the corresponding feature bundles were termed *object files* (Kahneman et al., 1992; Kahneman & Treisman, 1984). Such files include any perceptual features that had been activated concurrently. Perhaps counterintuitively, this notion suggests that feature binding should also include features of actions that had been performed in the face of a given stimulus, as action plans can be conceived as relating to perceptual features of the sensory consequences of a movement (Hommel, 2009; Kunde, 2001; Pfister, 2019; see also Henderson, 1996). This speculation has been confirmed by numerous empirical studies using behavioural (Frings et al., 2007; Hommel, 1998) or neurophysiological measures (Dobbins et al., 2004; Kleimaker et al., 2020; Pastötter et al., 2021). To highlight the notion that any perceptual or action event can be subject to binding and retrieval, current research has therefore adopted the term *event file* to describe what results from feature binding in the cognitive system (Hommel, 2004; Hommel et al., 2001).

The latest instalment of binding and retrieval accounts is the Binding and Retrieval in Action Control framework (BRAC; Frings et al., 2020). It specifies that any stimulus, response or effect feature can be integrated into an event file and is thus able to instigate retrieval of any corresponding feature. This interplay explains a surprising variety of experimental observations in such diverse paradigms as negative priming (Frings et al., 2015), conflict paradigms (Dignath et al., 2019), task switching (Schumacher & Hazeltine, 2016; Waszak et al., 2003), and others. Furthermore, the BRAC framework suggests that binding and retrieval are separate, distinct processes that can be subject to different moderators, both in a bottom-up and in a top-down fashion.

Despite their success in offering a parsimonious explanation for many empirical phenomena, theoretical accounts such as the BRAC framework are currently limited in that they do not allow for specifying when binding actually occurs. Also, even though they leave room for flexible inclusion of any perceivable feature into an event file, they do not predict what features actually get bound in any specific episode. Only recently has research provided a principled approach to these questions, as we discuss in the following.

### When does binding occur? And what actually gets bound?

It seems plausible to assume that binding is triggered by a response of the cognitive system. This response can be overt, such as a body movement to affect the environment, or it can be covert such as a visual scanpath in object perception or an internal classification response. It is less evident whether the episode ends with this response. In fact, evidence suggests that also events following a response enter bindings with a response and can instigate retrieval later on (Dutzi & Hommel, 2009; Herwig & Waszak, 2012; Hommel, 2005; Janczyk et al., 2012; Moeller et al., 2016). Current theoretical approaches have therefore suggested that it is not the response itself that completes a binding episode. Instead, they assume this process to depend on evaluating the action episode as successful—the *success-based account* (Hommel, 2005).

**Fig. 1:**
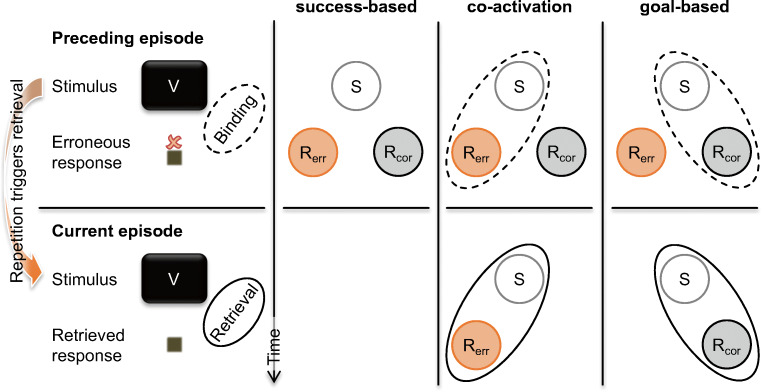
Theoretical accounts and corresponding predictions. Binding in a preceding episode as illustrated by dashed ellipses and retrieval in a current episode as illustrated through solid ellipses. Circles indicate feature codes that are active in the cognitive system at the time of error commission. Features of stimuli are denoted with S and a repetition of stimuli features in successive episodes retrieves any bound response features. Features of erroneous and correct responses are denoted as R_err_ and R_cor_, respectively. The success-based account assumes binding to be conditional on evaluating an action episode as successful, thus predicting binding and retrieval not to occur in the face of action slips. The co-activation account assumes binding of any feature that is sufficiently active, thus predicting binding and later retrieval of the erroneous response. The goal-based account assumes binding of the intended, correct response, thus predicting later retrieval of this response even if it had not actually been executed

The success-based account of binding was tacitly accepted in later work—perhaps because of a lack of contradicting observations, or because the account accommodates findings on episodic binding of events following a response (i.e., action effects) quite elegantly. Its major claim therefore was not critically examined until recently. Such critical testing seems warranted, though, because there are several logical alternatives to the idea of conceptualizing binding processes as being conditional on successful performance. We therefore proposed that studying binding and retrieval for action slips provides an ideal testbed to test the success-based account against two potential competitor accounts, because these accounts make specific predictions for such erroneous situations as sketched in Fig. 1 (Foerster et al., 2022a). For one, the cognitive system might simply bind all available features after a certain time following a response in an incidental manner— the *co-activation account*. In case of an unsuccessful response such as an action slip, this would yield binding of erroneous actions with corresponding stimulus and effect features (note that the term co-activation is also used in the literature on cognitive control with a distinct meaning; e.g., Bahle et al., 2020). Preliminary evidence for this claim is the observation of binding and retrieval for unsuccessful stopping responses (i.e., failures to cancel a correct response upon encountering a sudden stop signal; Giesen & Rothermund, 2014; for additional background on the stop-signal paradigm, see Logan & Cowan, 1984; Verbruggen & Logan, 2008). A third option is that binding integrates features of the intended correct response rather than features of the actually executed erroneous response in this situation—the *goal-based account*. This possibility receives preliminary support from theories on error processing that suggest the correct action to be represented quite strongly during error commission, giving rise to swift error corrections (Crump & Logan, 2013).

Findings for binding between features of stimuli and responses (i.e., stimulus–response binding) clearly favoured the goal-based account, suggesting that binding for action slips operates on codes of the intended, correct response rather than the erroneous response that had actually been executed (Exp. 1 in Foerster et al., 2022a; Foerster et al., 2021; Parmar et al., 2022). For binding between features of responses and their effects (i.e., response–effect binding), by contrast, results suggested co-activation-based bindings between the erroneous response and its following sensory effect (Exp. 2 in Foerster et al., 2022a). This pattern suggests that binding and retrieval are remarkably tuned to contingencies in the agent’s environment while at the same time promoting flexible and efficient action control. Based on these findings, the current study aimed at providing a thorough specification of the goal-based account: For one, we therefore examined the role of feedback. Second, we scrutinized the fate of the erroneous response when its features cannot enter bindings with any overt, direct consequences because such consequences are absent.

### Specifying the goal-based account

Goal-based binding between stimuli and the intended correct response, to date, has only been studied in paradigms where participants did not receive explicit feedback immediately after committing an error (Foerster et al., 2022a; Foerster et al., 2021). Research on error processing seems to back this initial choice of experimental design at first glance. Skilled typists, for instance, are able to detect, identify, and correct errors impressively well even when their hands are covered and when they are not able to see the text they produce either (Rabbitt, 1978). Nonexperts also corrected their errors promptly in reaction time tasks that did not provide error feedback (Fiehler et al., 2005; Rabbitt, 1966, 2002). These studies suggest that correct response tendencies emerge swiftly during error commission and an increasing activation of the correct response might be a precursor for goal-based binding. Explicit feedback for the erroneous response might boost such activation by triggering greater error awareness, thereby pulling attention away from representations of the erroneous response to the correct response more efficiently. As such, explicit error feedback might increase the efficiency of goal-based binding processes. At the same time, the presentation of feedback introduces a novel event between the establishment of a binding and later retrieval, which might reduce the observed effects (Hilchey et al., 2017; Spadaro et al., 2012). In two experiments, we therefore replicated effects of binding and retrieval of task-relevant stimuli to the intended correct response after action slips without explicit feedback (Experiment 1) and with explicit error feedback (Experiment 2).

Aside from corrective measures in terms of binding and retrieval and the immediate delivery of the correct response, errors themselves leave early traces in cognition and action, as, for example, in the form of a weaker and shorter execution of errors (Bode & Stahl, 2014; Hochman et al., 2017; Rabbitt, 1978; Śmigasiewicz et al., 2020) as well as of a pronounced negative deflection in electrophysiological data, the so-called error-related negativity, shortly after the error (Falkenstein et al., 2000; Gehring et al., 2012). Errors further affect following actions in the form of prolonged response times (RTs), prominently known as post-error slowing effect. This empirical phenomenon has been mainly attributed to maladaptive processes (e.g., pronounced orienting toward the error or intensified monitoring of the response and its effects) and to adaptive processes that shift behavior to a more conservative threshold (Crump & Logan, 2013; Dutilh et al., 2011; Jentzsch & Dudschig, 2009; Notebaert et al., 2009; Wessel, 2018). Together, these results suggest that cognitive processes provide immediate and sustained countermeasures that detect erroneous responses, bring them to a halt, and change the course of action toward success. An open question is whether these countermeasures also provoke a continued inhibition of the erroneous response or whether the execution of the erroneous response leaves it at an increased level of activation, either alone or in a compound with the correct response. Whereas Experiments 1 and 2 provided preliminary post hoc evidence for the hypothesis of continued activation, Experiment 3 provides a thorough test of this conjecture.

## Experiments 1 and 2

In Experiment 1, we replicated the setup of previous demonstrations of goal-based binding (Foerster et al., 2022a) without using feedback. In Experiment 2, we introduced explicit error feedback. We assessed whether feedback would boost effects of goal-based binding between relevant stimuli and intended correct responses through increased error awareness, or whether feedback would diminish binding and retrieval effects through the introduction of a new event between the binding episode and later retrieval cues. On the one hand, existing evidence demonstrated weaker goal-based binding and retrieval effects for relevant stimuli following an erroneous response than following a correct response (Foerster et al., 2022a). This weaker effect could be a consequence of overlooked errors in a subset of trials for which the executed erroneous response instead of the correct response would then enter a binding with the target. Explorative analyses of the distribution of the data did not support this explanation though because they neither pointed to bimodality (e.g., the presence of goal-based binding in only one part of the data and the absence of any binding or small reversed effects in the other part of the data) nor to correlations between binding and retrieval effects and effects of error processing (i.e., stronger binding and retrieval effects with stronger post-error slowing). Further, binding and retrieval effects between irrelevant stimuli and correct response observed in follow-up work were equivalent after an error compared with after a correct response (Foerster et al., 2021). The following two experiments put the impact of explicit knowledge about the error through feedback on this process to a direct test. At the same time, they provide the opportunity for a high-power replication of goal-based binding and retrieval between targets and intended correct responses.

The research design further allowed us to scrutinize the fate of the erroneous response by analyzing situations in which a previously committed error became the correct response as signalled by the next stimulus. Here, continued activation of the erroneous response would counter effects of post-error slowing that were routinely observed for repetitions of the correct response in both previous studies. Such a descriptive pattern was in fact present in the preceding studies though we had not scrutinized this (then unpredicted) pattern by targeted analyses.

Participants conducted a simple forced-choice response task in which they responded to letter stimuli with a left versus right keypress in a series of trials (Foerster et al., 2022a). Experiment 1 did not provide any feedback immediately after a commission error, while Experiment 2 did provide such feedback. We further took care to match the timing across both experiments to enable meaningful between-experiment comparisons. The target set comprised four letters (4:2 mapping). For both experiments, we analyzed performance as a function of preceding response (correct vs. error) and condition sequence (target repetition | correct response repetition vs. target change | correct response repetition vs. target change | correct response change). Replicating existing findings on goal-based binding and retrieval for relevant stimuli, we hypothesized that correct response repetitions (i.e., trial sequences in which both targets afford the same correct response) would be faster for target repetitions than for target changes after a correct response and crucially, also after an error (Foerster et al., 2022a). We further tested whether this effect would also emerge with error feedback in Experiment 2, and whether it would differ in magnitude (i.e., whether it would be reduced or amplified) compared with Experiment 1. The experiments were conducted in parallel with random allocation of participants to allow for a comparison of the feedback conditions.

### Method

The preregistration (osf.io/978ym), experimental program, data and the analyses syntax of both experiments are publicly available in a project repository (osf.io/u837c) at the Open Science Framework.

#### Participants

For determining the sample size, we considered effect sizes that we observed for differences in binding and retrieval effects of relevant stimuli and intended correct responses as well as correct response repetition benefits between correct and erroneous action episodes in our lab (*d*_z_ ≥ 0.77; Foerster et al., 2022a). A sample of 24 participants has a power of 95% to detect this effect size in a two-tailed paired-samples test with an alpha of 5% (calculated with the power.t.test function in R, Version 3.3.3). We did not have any indication on the size of a potential modulation of these effects by feedback. We therefore decided for a larger sample size of 48 participants per experiment, which allows to detect between-subject modulations of about *d*_s_ = 0.52 with the same specification as in the power analysis above. We allocated participants (80 female, 76 right-handers, *M*_*age*_ = 24 years, *SD*_*age*_ = 6 years) randomly to the two experiments of which one did (*n*_1_ = 48) and the other one did not present immediate error feedback (*n*_2_ = 48).

#### Apparatus and stimuli

Participants conducted the experiment on a setup with a 24-in. screen with a display resolution of 1,920 × 1,080 pixels and a refresh rate of 100 Hz. The laboratory room had five of these workstations for parallel data collection. Participants responded with the keys *F* and *J* on a standard QWERTZ keyboard with their index fingers to letter stimuli. We chose such simple alphanumeric stimuli because previous empirical data indicates that these stimuli would not introduce a confound in terms of perceptual priming effects (Pashler & Baylis, 1991). The target letters *T* and *N* were mapped to one key, and the target letters *V* and *K* were mapped on the other key, and we counterbalanced the assignment of letter pairs to response keys across participants. We further displayed additional irrelevant letters on screen by drawing one irrelevant letter per trial from a set of eight potential letters (i.e., *O*, *W*, *X*, *U*, *Z*, *Y, H*, *A*). These additional letters were intended to provoke commission errors through perceptual noise. Relevant and irrelevant letters appeared in white font against a black background in the centre of the screen with the target letter in the centre, surrounded by an irrelevant letter appearing above, below (vertical displacement: 65 px), left and right (horizontal displacement: 58 px) relative to the target.

#### Procedure

Written instructions introduced participants to the task with the mapping of target letters to response keys, emphasizing fast and accurate responses. Irrelevant letters were introduced as distractors that should be ignored. Participants had the chance to read through all instructions again if they preferred to. The first block served as practice and provided feedback about the accuracy of each response in both experiments. For all following experimental trials, we provided feedback only for omission errors (Experiment 1) or for omission and commission errors (Experiment 2). Our main goal was to provoke commission errors, so we aimed to nudge participants toward fast responding by presenting feedback for omission errors in both experiments. At the beginning of each block, participants received information about which feedback would be provided and were encouraged to respond as fast and accurately as possible. At the end of each block, participants received a summary of their performance, including average response times (RTs) of correct responses, the number of false responses (commission errors and keypresses with any other key than the instructed ones), and omissions.

**Fig. 2:**
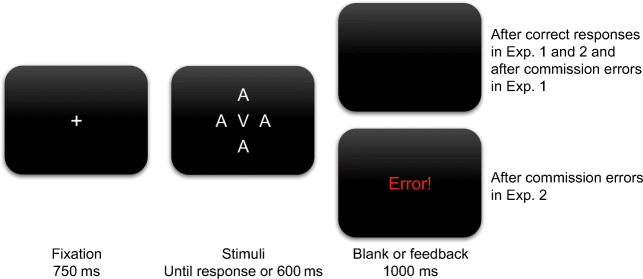
Trial procedure of Experiments 1 and 2. After fixation, participants were to respond to the central target letter within 600 ms with one of two response keys. The four irrelevant letters had to be ignored and did not map to the two response keys. In experimental trials, a correct response triggered a blank screen in both experiments whereas a commission error triggered a blank screen in Experiment 1 but an error message in Experiment 2. Both the blank screen and the error feedback appeared for 1,000 ms

Figure 2 provides an overview of the trial procedure. A trial began with a fixation for 750 ms, followed by the presentation of the target letter and irrelevant letters. These stayed on screen until participants responded or for a maximum of 600 ms, i.e., there was a response deadline of 600 ms. There was either a blank screen or feedback for 1,000 ms before the next trial commenced. If participants did not deliver a response on time (i.e., omission error), the feedback “Too slow!” (German: “Zu langsam!”) appeared in red font. A commission error or a response with any other key than the instructed ones produced the error message “Error!” (German: “Fehler!”) in practice blocks of both experiments and in experimental blocks of Experiment 2, whereas only a blank screen appeared in experimental blocks of Experiment 1. After a correct response, the feedback “Good!” (German: “Gut!”) appeared in green font in practice blocks and a blank screen in experimental blocks of both experiments.

**Fig. 3:**
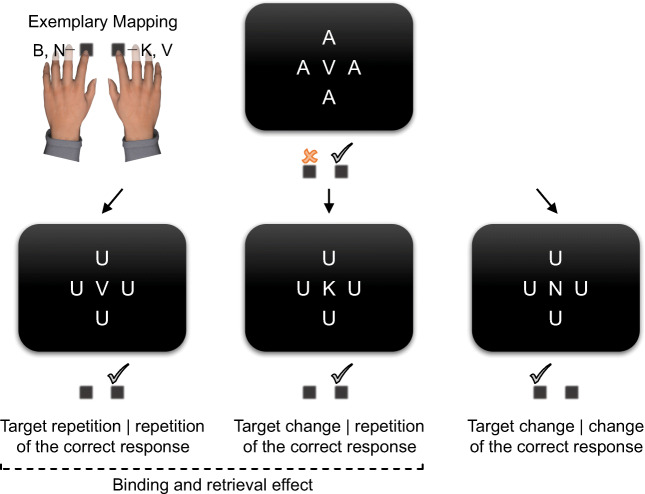
Experimental conditions of Experiments 1 and 2. We examined trial sequences with a correct response or commission error in the preceding trial. The mapping of four target letters to two response keys resulted in three condition sequences. Target repetitions came with a repetition of the correct response (left) and target changes either came with a repetition of the correct response (middle) or a change of the correct response (right). Binding and retrieval effects are assessed via the comparison of correct response repetitions between target repetitions and changes, whereas changes of the correct response allow for preliminary conclusions about the representation of the erroneous response in case of goal-based binding

One practice block and 19 experimental blocks featured 56 trials each. One of the four target letters was chosen randomly for the first trial of each block. In all following trials, we opted for a pseudorandom strategy for target selection. We created two arrays that each held a random sequence of three instances of each of the three conditions sequences (i.e., target repetitions | correct response repetitions, target changes | correct response repetitions, and target changes | correct response changes; see Fig. 3). After a commission error in the preceding trial, the current condition sequence was drawn from one array. For all other response types in the preceding trial, the current condition sequence was drawn from the second array. The selected condition sequence then determined the current target. We implemented this selection strategy to collect sufficiently many observations for each condition sequence even after rare errors. An array was randomized whenever all its elements had been drawn once. In case of correct response changes, one of the two possible targets was drawn randomly. A third array held a random sequence of the eight irrelevant stimuli. Whenever all irrelevant stimuli had been drawn once, the array was randomized with the restriction that two successive trials would not present the same irrelevant stimulus to control for potential binding and retrieval effects for irrelevant stimuli (Foerster et al., 2021; Frings et al., 2007).

#### Data treatment and analysis

We did not analyze the practice block. We could also not analyze the first trial of each block because we investigated performance as a function of the preceding response. We further excluded trials with a miscellaneous error (i.e., random key press that did not match the two instructed keys or key presses during fixation or blank/feedback; 2.8%) or with an omission error (4.7%) in the preceding trial. This selection of trials went into our analysis of the percentage of commission errors and omission errors, computed as the number of commission (omission) errors divided by the sum of correct responses and commission (omission) errors. For the remaining analyses, we also excluded any erroneous response (14.5%) and excluded the remaining correct trials as outliers separately for each participant if their RT deviated more than 2.5 standard deviations from the respective cell mean (i.e., preceding response × condition sequence; 1.2%). Five participants in Experiment 1 and four participants in Experiment 2 provided less than ten observations in at least one of the design cells after these exclusions and were excluded from all statistical analyses.

As our main analysis, we analyzed correct RTs of each experiment as a function of preceding response (correct vs. error) and condition sequence (target repetition | correct response repetition vs. target change | correct response repetition vs. target change | correct response change) in 2 × 3 within-subjects analyses of variance (ANOVAs). In case of significant two-way interactions, we scrutinized binding and retrieval effects in 2 × 2 ANOVAs (excluding target change | correct response change trials). A significant interaction in this ANOVA was followed up by two-tailed paired-samples *t*-tests, testing binding and retrieval effects separately for correct and erroneous action episodes. We analyzed commission rate and omission rate in the same way to address potential speed–accuracy trade-offs. We further assessed RT differences between preceding correct and erroneous responses for trial sequences with changes of the correct response in exploratory two-tailed paired-samples *t*-tests to gather preliminary evidence on the representation of the erroneous response.

Additional analyses targeted the relative variance of RTs (RV_RT_; $\frac{SD(RT)\ast SD(RT)}{M(RT)}$) and the difference in response durations (ΔRD) between successive trials following the same analysis plan. If there is goal-based binding and retrieval of the temporal features of a response, this should be reflected in similar effects as for RTs.

Finally, we compared the findings of each individual experiment between both experiments by adding the between-subjects factor feedback (absent vs. present) to the ANOVAs. We will only report main effects of feedback and interactions with feedback for brevity for this latter analysis. For the omnibus ANOVAs with the three-levelled factor condition sequence, we report Greenhouse–Geisser corrections with ε estimates in case of violations of sphericity. We provide the effect size *d*_*z*_ ($\frac{M\left(\Delta RT\right)}{SD\left(\Delta RT\right)}$) for each *t*-test. Descriptive statistics for all dependent variables for each experimental cell are provided in the Appendix, in Table 1 for Experiment 1 and in Table 2 for Experiment 2.

As in our preceding study, we conducted exploratory correlational and distributional analyses to get a better understanding of differences in binding and retrieval effects between correct and erroneous action episodes (Foerster et al., 2022a). To maximize power for these analyses, we conducted these analyses on the pooled data of both experiments here. We correlated binding and retrieval effects of correct and erroneous action episodes in RTs, percentage of commission error and omission errors. We also correlated binding and retrieval effects in RTs with the differences scores in RTs of preceding erroneous and preceding correct responses (i.e., post-error slowing effects). All reported tests were two-tailed. For distributional analyses, we report bimodality coefficients (Freeman & Dale, 2013; Pfister et al., 2013; SAS Institute Inc, 1990) of binding and retrieval effects in RTs for preceding correct and erroneous responses. We also compared bimodality coefficients and skewness parameters of the individual RT data of each participant for trial sequences with repetitions of the target and the correct response between preceding correct and preceding erroneous responses in paired-samples *t*-tests.

### Results of Experiment 1

#### RTs

**Fig. 4:**
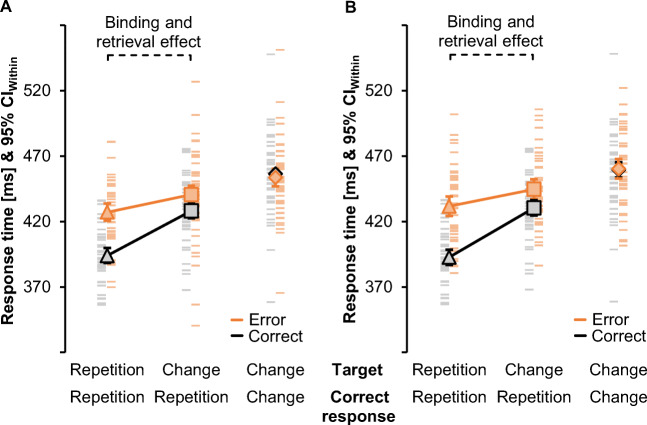
Main results of Experiments 1 and 2. Response times with sample means (triangles, squares and diamonds) and individual values (lines) for Experiment 1 without feedback after commission errors (**A**) and for Experiment 2 with such feedback (**B**). Both feedback conditions showed the expected binding and retrieval effect in faster repetitions of the correct response if the target repeated (triangle) relative to target changes (square) after a preceding correct action episode (grey/black) and after a preceding commission error (bright orange). Response times did not differ when the correct response changed (diamond) to the response that had previously been given in error (bright orange) or to the neutral response after a correct episode (grey/black). Error bars represent 95% within-subject confidence intervals for the full factorial design (CI_Within_; Loftus & Masson, 1994)

Figure 4a shows RTs for the main analysis. A preceding erroneous response prolonged RTs compared with a preceding correct response, *F*(1, 42) = 31.91, *p* < .001, η_p_^2^ = .43. Condition sequence also impacted RTs, *F*(2, 84) = 80.21, *p* < .001, η_p_^2^ = .66 (ε = .83), with the fastest responses for sequences with target repetitions and correct response repetitions and slowest responses for target changes and correct response changes. The two factors interacted, *F*(2, 84) = 29.69, *p* < .001, η_p_^2^ = .41. The reduced ANOVA also showed post-error slowing, *F*(1, 42) = 50.03, *p* < .001, η_p_^2^ = .54, and faster correct response repetitions for target repetitions than target changes, *F*(1, 42) = 68.52, *p* < .001, η_p_^2^ = .62, as well as a significant two-way interaction, *F*(1, 42) = 19.17, *p* < .001, η_p_^2^ = .31. Binding and retrieval effects were larger after a correct response, *t*(42) = 12.51, *p* < .001, *d*_*z*_ = 1.91, than after an error, *t*(42) = 2.98, *p =* .005, *d*_*z*_ = 0.45.

For correct response changes, RTs did not differ between preceding correct and preceding erroneous responses, *t*(42) = −0.90, *p =* .373, *d*_*z*_ = −0.14.

#### Percentage of commission errors

The percentage of commission errors did not differ between preceding responses, *F* < 1. Condition sequences had a significant impact, *F*(2, 84) = 23.70, *p* < .001, η_p_^2^ = .36 (ε = .66), with the smallest percentage of commission errors in trials with target repetitions and correct response repetitions, followed by trials with target changes and correct response repetitions and the highest values for trials with target changes and correct response changes. The two-way interaction was also significant, *F*(2, 84) = 26.08, *p* < .001, η_p_^2^ = .38. The reduced ANOVA showed that the percentage of commission errors was higher after a preceding error than after a correct response *F*(1, 42) = 6.92, *p* = .012, η_p_^2^ = .14. There were also more commission errors for sequences where correct responses repeated if targets changed than if targets repeated, *F*(1, 42) = 34.03, *p* < .001, η_p_^2^ = .45. The interaction was also significant *F*(1, 42) = 38.73, *p* < .001, η_p_^2^ = .48, indicating that binding and retrieval effects were significant after a preceding correct response, *t*(42) = 13.71, *p* < .001, *d*_*z*_ = 2.09, but not after a preceding erroneous response, *t*(42) = -0.32, *p* = .754, *d*_*z*_ = −0.05.

#### Percentage of omission errors

Participants omitted more responses after an erroneous than after a correct response, *F*(1, 42) = 13.61, *p* = .001, η_p_^2^ = .25. There was also a significant main effect of condition sequence, *F*(2, 84) = 19.97, *p* < .001, η_p_^2^ = .32 (ε = .80), pointing to lower percentages of omissions for sequences with target repetitions and correct response repetitions than for sequences with target changes and correct response repetitions and highest values for sequences with target changes and correct response changes. The interaction of both factors was not significant *F*(2, 84) = 1.44, *p* = .242, η_p_^2^ = .03. The reduced ANOVA showed that percentages of omissions were higher after a preceding error than after a preceding correct response, *F*(1, 42) = 8.72, *p* = .005, η_p_^2^ = .17, and if targets changed than repeated while correct responses repeated, *F*(1, 42) = 18.81, *p* < .001, η_p_^2^ = .31. The interaction of both factors was not significant, *F*(1, 42) = 3.25, *p* = .078, η_p_^2^ = .07.

#### RV_RT_

The RV_RT_ was lower after correct than after erroneous responses, *F*(1, 42) = 14.13, *p* = .001, η_p_^2^ = .25, and it also varied with condition sequence, *F*(2, 84) = 12.62, *p* < .001, η_p_^2^ = .23 (ε = .59), with highest values for target changes and correct response repetitions, followed by target repetitions and correct response repetitions and lowest values for target changes and correct response changes. The interaction of both factors was significant, *F*(2, 84) = 6.56, *p* = .005, η_p_^2^ = .14 (ε = .76). The reduced ANOVA again showed a lower RV_RT_ after a preceding correct than after a preceding erroneous response, *F*(1, 42) = 11.21, *p* = .002, η_p_^2^ = .21, and after a target repetition than target change for correct response repetitions, *F*(1, 42) = 16.93, *p* < .001, η_p_^2^ = .29. The interaction was significant, *F*(1, 42) = 11.41, *p* = .002, η_p_^2^ = .21, indicating that binding and retrieval effects were only evident after a correct response, *t*(42) = 10.25, *p* < .001, *d*_*z*_ = 1.56, but not after an error, *t*(42) = 0.09, *p* = .932, *d*_*z*_ = 0.01.

#### ΔRD

ΔRDs were smaller after a preceding correct than after a preceding erroneous response, *F*(1, 42) = 41.65, *p* < .001, η_p_^2^ = .50, whereas the main effect of condition sequence was not significant, *F* < 1. The interaction of both factors was significant, *F*(2, 84) = 27.17, *p* < .001, η_p_^2^ = .39 (ε = .76). The reduced ANOVA only showed smaller ΔRDs after a correct response than after an error, *F*(1, 42) = 59.54, *p* < .001, η_p_^2^ = .59. The main effect of condition sequence and the interaction were not significant, *F*s ≤ 2.61, *p*s ≥ .113, η_p_^2^ ≤ .06.

### Results of Experiment 2

#### RTs

Figure 4b shows the pattern of RTs for the main analysis. RTs were longer after a preceding erroneous than correct response, *F*(1, 43) = 62.00, *p* < .001, η_p_^2^ = .59. There was a significant main effect of condition sequence, *F*(2, 86) = 119.38, *p* < .001, η_p_^2^ = .74 (ε = .83), again with the fastest responses for trial sequences with target repetitions and correct response repetitions and slowest responses for target changes and correct response changes. The interaction of both factors was significant, *F*(2, 86) = 24.56, *p* < .001, η_p_^2^ = .36 (ε = .69). The reduced ANOVA indicated slower responses after an error than after a correct response, *F*(1, 43) = 56.83, *p* < .001, η_p_^2^ = .57, and for correct response repetitions with target changes than with target repetitions, *F*(1, 43) = 104.64, *p* < .001, η_p_^2^ = .71. The interaction of both factors was significant, *F*(1, 43) = 54.71, *p* < .001, η_p_^2^ = .56, indicating that binding and retrieval effects were larger after a correct response, *t*(43) = 13.29, *p* < .001, *d*_*z*_ = 2.00, than after an error, *t*(43) = 4.15, *p* < .001, *d*_*z*_ = 0.63.

Post-error slowing was not significant for correct response changes, *t*(42) = 0.17, *p =* .867, *d*_*z*_ = 0.03.

#### Percentage of commission errors

Whether the preceding response was correct or erroneous did not affect the percentage of commission errors, *F* < 1. There was a significant main effect of condition sequences, *F*(2, 86) = 30.04, *p* < .001, η_p_^2^ = .41 (ε = .88), with the smallest amount of commission errors in trial sequences with target repetitions and correct response repetitions, followed by target changes and correct response repetitions and the highest values for target changes and correct response changes. The two-way interaction was significant, *F*(2, 86) = 15.70, *p* < .001, η_p_^2^ = .27 (ε = .87). The reduced ANOVA showed an increased percentage of commission errors after a preceding error than after a correct response *F*(1, 43) = 6.33, *p* = .016, η_p_^2^ = .13. There were also more commission errors for target changes than for target repetitions in correct response repetition trials, *F*(1, 43) = 36.74, *p* < .001, η_p_^2^ = .46. The interaction was also significant *F*(1, 43) = 29.06, *p* < .001, η_p_^2^ = .40, indicating that binding and retrieval effects were significant after a preceding correct response, *t*(43) = 9.09, *p* < .001, *d*_*z*_ = 1.37, but not after a preceding erroneous response, *t*(43) = 0.34, *p* = .735, *d*_*z*_ = 0.05.

#### Percentage of omission errors

The percentage of omission errors was higher after a preceding erroneous than after a preceding correct response, *F*(1, 43) = 85.15, *p* < .001, η_p_^2^ = .66. Condition sequence also had a significant impact, *F*(2, 86) = 14.52, *p* < .001, η_p_^2^ = .25 (ε = .80), with the lowest percentage of omission errors for target repetitions and correct response repetitions, followed by target changes and correct response repetitions and highest values for target changes and correct response changes. The interaction of both factors was significant, *F*(2, 86) = 3.96, *p* = .023, η_p_^2^ = .08. The reduced ANOVA showed increased percentages of omission errors after an error than after a correct response, *F*(1, 43) = 41.48, *p* < .001, η_p_^2^ = .49, and for correct response repetitions with target changes than with target repetitions, *F*(1, 43) = 13.58, *p* = .001, η_p_^2^ = .24. The interaction of both factors was significant, *F*(1, 43) = 9.53, *p* = .004, η_p_^2^ = .18, indicating significant binding and retrieval effects after a preceding correct response, *t*(43) = 5.60, *p* < .001, *d*_*z*_ = 0.84, but not after a preceding error, *t*(42) = 0.39, *p* = .697, *d*_*z*_ = 0.06.

#### RV_RT_

A preceding correct response led to a lower RV_RT_ than a preceding erroneous response, *F*(1, 43) = 28.29, *p* < .001, η_p_^2^ = .40. There was also a significant main effect of condition sequence, *F*(2, 86) = 15.19, *p* < .001, η_p_^2^ = .26 (ε = .72), with highest values for trial sequences with target changes and correct response repetitions, followed by target repetitions and correct response repetitions and lowest values for target changes and correct response changes. The interaction of both factors was significant, *F*(2, 86) = 8.20, *p* = .001, η_p_^2^ = .16. The reduced ANOVA indicated that the RV_RT_ was lower after a preceding correct than after a preceding erroneous response, *F*(1, 43) = 17.67, *p* < .001, η_p_^2^ = .29, and for target repetitions than target changes with correct response repetitions, *F*(1, 43) = 22.61, *p* < .001, η_p_^2^ = .35. The interaction was significant, *F*(1, 43) = 25.62, *p* < .001, η_p_^2^ = .37, indicating that binding and retrieval effects were only evident after a correct response, *t*(43) = 9.02, *p* < .001, *d*_*z*_ = 1.36, but not after an error, *t*(42) = −0.02, *p* = .983, *d*_*z*_ < 0.01.

#### ΔRD

Mean ΔRDs were smaller after a correct response than after an erroneous response, *F*(1, 43) = 123.43, *p* < .001, η_p_^2^ = .74. The main effect of condition sequence was not significant, *F* < 1 but the interaction was significant, *F*(2, 86) = 50.14, *p* < .001, η_p_^2^ = .54 (ε = .76). The reduced ANOVA showed again smaller ΔRDs after a correct response than after an error, *F*(1, 43) = 181.11, *p* < .001, η_p_^2^ = .81. The main effect of condition sequence and the interaction were not significant, *F* < 1.

### Results of the between-experiment comparisons

#### RTs

There was neither a significant main effect of feedback nor any interaction involving feedback in RTs, *F*s ≤ 1.18, *p*s ≥ .280, η_p_^2^ ≤ .01.

#### Percentage of commission errors

Neither the main effect of feedback nor any of the interactions including feedback were significant, *F*s ≤ 1.34, *p*s ≥ .251, η_p_^2^ ≤ .02.

#### Percentage of omission errors

The main effect of feedback and the interactions were not significant, *F*s ≤ 2.37, *p*s ≥ .127, η_p_^2^ ≤ .03.

#### RV_RT_

The main effect of feedback was not significant, *F*(1, 85) = 1.22, *p* = .273, η_p_^2^ = .01. The interaction of feedback and preceding response was significant, *F*(1, 85) = 3.98, *p* = .049, η_p_^2^ = .05, indicating that the post-error variability increase was numerically larger (but came with smaller effect sizes) without feedback in Experiment 1, *t*(42) = 3.76, *p* = .001, *d*_*z*_ = 0.57, than with feedback in Experiment 2, *t*(43) = 5.32, *p* < .001, *d*_*z*_ = 0.80. The two-way interaction of feedback and condition sequence and the three-way interaction were not significant, *F*s ≤ 2.46, *p*s ≥ .101, η_p_^2^ ≤ .03.

#### ΔRD

Neither the main effect of feedback nor any of the interactions including feedback were significant, *F*s ≤ 1.10, *p*s ≥ .298, η_p_^2^ ≤ .01.

### Results of explorative pooled analyses

Binding and retrieval effects of correct and erroneous action episodes correlated significantly for RTs, *r* = .28, *t*(85) = 2.66, *p* = .009, but not in percentages of commission errors, *r* = −.02, *t*(85) = 0.16, *p* = .877, or percentages of omission errors, *r* = .13, *t*(85) = 1.25, *p* = .215. There were no significant correlations of post-error slowing with binding and retrieval effects in RTs after a correct response, *r* = .07, *t*(85) = 0.65, *p* = .518, and after an erroneous response, *r* = .17, *t*(85) = 1.55, *p* = .124. The bimodality coefficients of binding and retrieval effects in RTs of preceding correct (BC = .374) and preceding erroneous responses (BC = .230) were both below the critical value of .555, indicating unimodality. Responses in trials with target repetitions and correct response repetitions were more skewed, *t*(86) = 7.12, *p* < .001, *d*_*z*_ = 0.76, and less unimodal, *t*(86) = 2.59, *p* = .011, *d*_*z*_ = 0.28, after a correct response (*M*_*skewness*_ = 0.535*, SD*_*skewness*_ = 0.234; *M*_*BC*_ = 0.451*, SD*_*BC*_ = 0.056*)* than after a preceding erroneous response (*M*_*skewness*_ = 0.213*, SD*_*skewness*_ = 0.443; *M*_*BC*_ = 0.424*, SD*_*BC*_ = 0.082*)*.

### Discussion

Experiment 1 and 2 investigated goal-based binding and retrieval between relevant stimuli and intended correct responses for commission errors that were not or were immediately fed back to participants. Replicating our preceding findings (Foerster et al., 2022a), RTs were faster for target repetitions than target changes that came with a repetition of the correct response; this effect was observed after correct responses and also (albeit smaller) after errors. The latter effect supports the goal-based approach to binding for action slips.

Binding and retrieval emerged equally strong across feedback conditions. The presence of feedback neither modulated this effect itself, nor the difference of the effect after an erroneous compared with a correct response. Feedback only had an impact on the post-error increase in RT variability. For both feedback conditions, binding and retrieval effects were weaker after an error than after a correct response, although explicit error feedback should have enabled participants to detect each error. These results strongly suggest that the modulation of the strength of binding and retrieval effects through the preceding response is not a by-product of a lack of error awareness (e.g., in a subset of trials). In line with that, the distributional analyses pointed to unimodal distributions of binding and retrieval effects and RTs in trials with target repetitions and correct response repetitions as well as absent correlations between markers of error processing and binding and retrieval, also replicating previous distributional patterns (Foerster et al., 2022a). If participants missed some errors, resulting in bindings between the target and the executed erroneous responses, these analyses should have pointed to bimodal distributions and substantial correlations between binding and retrieval effects and effects of error processing.

Binding and retrieval effects were assessed through the comparison of trial sequences that featured correct response repetitions to target repetitions and to target changes. The third condition sequence with changes of both target and correct response could not inform our understanding of binding and retrieval because it lacks sequences with a change of the correct response but a target repetition as a baseline. However, this third condition allows for preliminary insights into the fate of the erroneous response. The result pattern shown in Fig. 4 suggests that post-error slowing effects were absent and descriptively even reversed when the current target stimulus called for the response that the participant had just given in error. The absence of post-error slowing suggests an increased and continued activation of the erroneous response. This conclusion is preliminary, however, because the design of Experiments 1 and 2 only featured two different responses. Each change of the correct response after error commission therefore coincided with a change to the erroneous response, without including a suitable control condition. Subjective expectations about the repetition rather than a change of responses might further drive these effects (Soetens et al., 1985), especially since correct response repetitions appeared in two-thirds of the trials in Experiment 1 and 2 and in our previous study to arrive at an equal proportion of random condition sequences (Foerster et al., 2022a). Experiment 3 therefore provides a more conclusive test of the representation of the erroneous response itself after goal-based binding.

## Experiment 3

As argued in the introduction, previous theoretical considerations and empirical investigations on error processing hint at an inhibition of the erroneous response (e.g., Rabbitt, 1978; Wessel, 2018). This theoretical stance seems to be at odds with the pattern observed in Experiments 1 and 2, as the results tentatively pointed to a continued high accessibility of the erroneous response instead, as suggested by absent post-error slowing effects for changes of the correct response.

Testing whether the erroneous response is inhibited, remains highly active, or rather quickly returns to a baseline activation requires a larger target and response set to introduce suitable baseline conditions. With a mapping of six letters to three responses, sequential analyses of RTs of correct responses that follow a commission error in the preceding trial can easily disentangle these three alternatives. In particular, a target change after an erroneous response either comes with a change of the correct response to the preceding erroneous response or to the neutral response option (i.e., this response neither corresponds to the preceding correct response nor to the preceding erroneous response). The comparison of these two condition sequences therefore allows to assess the accessibility of the former erroneous response. Inhibition of the erroneous response should render this response less accessible, leading to faster responding when changing to the neutral response as compared with changing to the preceding erroneous response. In contrast, a lingering activation of the erroneous response should increase its accessibility, leading to faster responding whenever the preceding erroneous response is correct. Rapid decay of the corresponding features, and thus a return to baseline activation should produce similar accessibility reflected in comparable RTs for preceding erroneous and neutral response options.

The described setup further allows to probe for an additional, provocative hypothesis on the fate of the erroneous response which may become bound to the intended correct response. Examinations on successful action episodes provided ample evidence that two individually planned and executed responses to different target stimuli can be bound together (Moeller & Frings, 2019a, 2019b, 2021). Later planning and execution of one of the responses retrieves any associated responses, facilitating their execution. We hypothesize that there also might be binding between the intended but not executed correct response and the erroneous response. If there is such binding, we assume that the preparation of one of the responses as the correct response in a following trial could retrieve the other one, increasing the likelihood of executing the retrieved response erroneously. Trial sequences with two successive commission errors where the target always changes and the correct response either repeats, changes to the preceding erroneous response or changes to the preceding neutral response offer an appropriate testbed for this prediction. Binding between the executed erroneous and the intended correct response leads to several predictions for the identity of commission errors in the upcoming trial depending on condition sequences. Commission errors in a trial sequence with a repetition of the correct response should match their preceding errors more frequently than their preceding neutral response options. Similarly, there should be more commission errors that match the preceding correct than neutral response option in trial sequences with correct response changes to the preceding erroneous response. Trial sequences with correct response changes to the preceding neutral response option should not show any effects of these binding mechanisms because there should not emerge any retrieval. In addition to these binding and retrieval effects, a residual activation of the erroneous response option predicts increased error commissions that match the preceding error (relates to trial sequences with either a repetition of correct responses or a change to the preceding neutral response option). In contrast, inhibition of the erroneous response should lead to opposite effects with less error commissions that match the preceding error whereas a quick decay of activation again predicts no differences here.

Finally, Experiment 3 provided the opportunity to replicate effects of goal-based binding and retrieval of stimuli and responses although the design provided less cell observations for this test. Therefore, we only approached these effects via RTs and percentages of commission errors.

### Method

The preregistration (osf.io/e65h7), experimental program, data, and the analyses syntax are also publicly available in a common project (osf.io/t25nb) at the Open Science Framework.

#### Participants

For this experiment, we had no indication of a potential effect size for our main research question. We required multiples of six participants to counterbalance our design factors. A sample size of 36 participants detects effects of *d*_*z*_ = 0.50 with a power of about 83% (alpha = 5%) in a two-tailed paired-samples *t*-test (calculated with the power.t.test function in R, Version 4.0.3). We therefore collected data and replaced participants with unusable data as specified in our preregistration until we had 36 participants for our main analysis (*M*_*Age*_ = 24 years, *SD*_*Age*_ = 6 years; 31 right-handed, five left-handed; 18 male, 15 female, three did not provide their gender). Fifty-seven participants conducted the whole experiment, and another three participants conducted only part of the experiment and aborted it early.

#### Apparatus and stimuli

We conducted the study online, hosted on Pavlovia, and used Prolific for participant recruitment. We only invited participants who had stated in Prolific that they were fluent in English. Participants could only participate if they stated that they had a desktop PC and we additionally told them to only participate with a minimum screen resolution of 800 × 600 pixels and a QWERTY-based keyboard, which we did not control, however. Participants responded with their index, middle and ring finger of their right hand via the three adjacent arrow keys (i.e., left, down and right). The target letters *B* and *N* mapped to one key, *F* and *T* to a second key and *K* and *V* to a third key. We counterbalanced the assignment of pairs of target letters to the three keys across participants. As in the preceding experiments, we presented additional irrelevant letters to increase perceptual noise (i.e., *C, H, M, P, S,* and *Y*). We reduced the number of irrelevant letters from eight to six to be able to present them in a balanced frequency in each block as in the former experiments. The letters appeared in white font against a black background in the centre of the screen. We created a 3 × 3 grid, consisting of eight instances of one irrelevant letter surrounding a central target letter. We displayed this letter grid in a fixed-width font.

#### Procedure

Participants first learned that they would have to respond to letters as fast and correct as possible via keypresses on their keyboard. To motivate them to conduct the whole experiment, we notified participants that the task would be challenging because of its high pace and encouraged them to try to keep on getting better. We then informed participants which fingers and keys they would use via text and in a graphical illustration that depicted the index, middle and ring finger of the right hand on the arrow keys. We then instructed them about the assignment of target letters to the three response keys via text and added this information in the graphical illustration of fingers and keys. Next, we informed them about the presence of additional distractor letters with an exemplary compound of target and distractor letters. We asked them to try their best to not get distracted by the additional letters.

At the beginning of each block, we again presented the illustration of the assignment of target letters to fingers and keys. At the end of each block, participants received a reminder of this assignment via text, and they received feedback about their performance as in the preceding experiments. At the beginning of the practice block, we informed participants that this block was considered practice and that they would receive feedback for each response. After they had finished the practice block, we informed participants that they would only receive feedback if they failed to respond in a trial from now. Before each experimental block, we encouraged participants to respond as fast and accurate as possible and to focus on the task throughout the whole block of trials while they should only take a rest between blocks.

The trial procedure was very similar to the preceding experiments except that there was no blank screen after responses, and that letters stayed on screen for an increased response deadline of 700 ms. We provided English error messages as per our translations in the preceding experiments, except that we fed back “Please use the arrow keys that point left, down, and right” if participants pressed any other than the instructed arrow keys in the practice block.

**Fig. 5:**
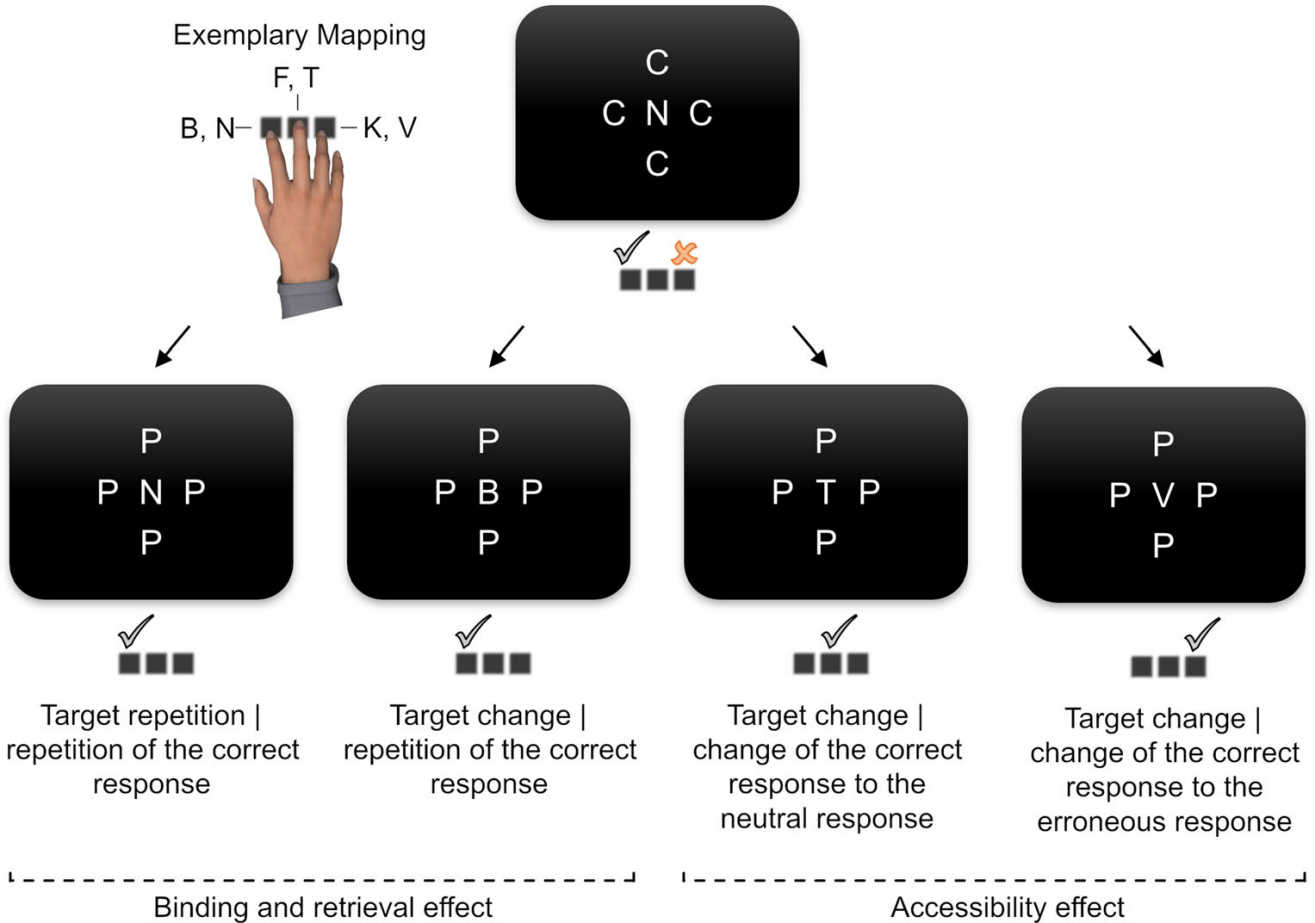
Experimental conditions of Experiment 3. We examined trial sequences with a correct response or commission error in the preceding trial for the replication of binding and retrieval effects and only trial sequences with a commission error in the preceding trial to assess the accessibility of the erroneous response. The mapping of six target letters to three response keys resulted in four condition sequences. Target repetitions came with a repetition of the correct response. Target changes either came with a repetition of the correct response, or with a change of the correct response to the neutral response or to the erroneous responses (the latter condition could only emerge after a preceding erroneous but not after a preceding correct response). Binding and retrieval effects after a correct response and after a commission error are assessed via the comparison of correct response repetitions between target repetitions and changes. Accessibility effects of the preceding erroneous response are assessed via the comparison of both conditions with a target change and a change of the correct response

The practice block featured 24 trials and the 19 experimental blocks featured 60 trials each—that is, four and 10 instances, respectively, of each target letter in a random sequence. The random sequence of targets should produce target repetitions with correct response repetitions and target changes with correct response repetitions each in about one out of six trials (see Fig. 5). The remaining four in six trials should feature an about equal number of target changes and correct response changes. An independent array held a random sequence of the six irrelevant stimuli. The array was randomized as soon as all irrelevant stimuli had been drawn once, with the restriction that successive trials would not present the same irrelevant stimulus to control for potential binding and retrieval effects of relevant and irrelevant stimuli (with responses).

#### Data treatment and analysis

We first excluded the practice block. We then computed the percentage of correct trials for each participant. Twenty participants responded correctly in less than 55% of the trials and were therefore excluded and replaced. We further excluded the first trial of each block for all remaining participants.

Our main analysis concerned the comparison of RTs of correct responses after a preceding commission error between condition sequences with a target change and a change of the correct response to the preceding neutral vs. erroneous response. We will refer to this comparison as the accessibility effect. RTs were excluded as outliers from this analysis if they deviated more than 2.5 standard deviations from the corresponding cell mean. One participant delivered less than 10 observations in at least one of the two cells and was therefore excluded and replaced. This selection of the sample was also applied in all other analyses.

We analyzed the percentage of commission errors (computed as number of commission errors relative to the sum of the number of commission errors and correct trials) after a preceding commission error in three paired-samples *t*-tests. First, we compared percentages of commission errors that matched the preceding erroneous or neutral response option for condition sequences where the target changed and the correct response repeated. Second, we compared percentages of commission errors that matched the preceding correct or neutral response option for condition sequences where the target changed and the correct response changed to the preceding erroneous response. Third, we compared percentages of commission errors that matched the preceding erroneous or correct response option for condition sequences where the target changed and the correct response changed to the preceding neutral response option.

Finally, our replication analysis targeted S–R binding in RTs and percentages commission errors. RTs were excluded as outliers if they deviated more than 2.5 standard deviations from the corresponding cell mean. We anticipated fewer observations for the replication analysis because trials with correct response repetitions were rare by design. We therefore employed a more liberal criterion for these analyses and included participants with at least five observations in the respective experimental cells. We did not replace participants that had to be excluded for this analysis. We provide descriptive statistics for these analyses in Table 3, in the Appendix.

### Results

#### Accessibility effects on RTs

**Fig. 6:**
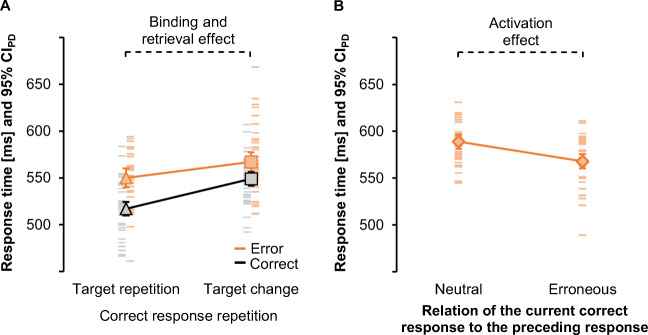
Main results of Experiment 3. Response times with sample means (triangles, squares and diamonds) and individual values (lines) for Experiment 3. **A** Binding and retrieval effects as tested by comparing correct response repetitions to a target repetition or to a target change after a preceding commission error (bright orange) and after a preceding correct response (grey/black). **B** Residual activation of the erroneous response as tested by comparing correct responses that corresponded either to a preceding neutral or to a preceding erroneous response after a preceding commission error (bright orange). Error bars represent 95% confidence intervals of paired differences (CI_PD;_ Pfister & Janczyk, 2013)

Figure 6b shows that participants responded faster after a commission error if the correct response changed to the preceding erroneous response (*M* = 568 ms*, SD* = 27 ms) than to the neutral response (*M* = 589 ms, *SD* = 23 ms), *t*(35) = 5.41, *p* < .001, *d*_*z*_ = 0.90.

#### Accessibility effects and response-response binding effects on percentages of commission errors

If the target changed and the correct response repeated, commission errors that matched the preceding erroneous response (*M* = 16.2%, *SD* = 9.8%) or the neutral response (*M* = 14.0%, *SD* = 10.0%) emerged equally often, *t*(35) = 1.26, *p* = .215, *d*_*z*_ = 0.21. If the target changed and the correct response changed to the preceding erroneous response, percentage of commission errors that matched the preceding correct response (*M* = 12.7%, *SD* = 8.7%) or the neutral response (*M* = 12.3%, *SD* = 9.0%) did also not differ in frequency, *t*(35) = 0.38, *p* = .704, *d*_*z*_ = 0.06. There was also no significant difference between percentages of commission errors that matched the preceding erroneous response (*M* = 13.6%, *SD* = 8.1%) or the correct response (*M* = 12.0%, *SD* = 8.4%) when the target changed and the correct response changed to the preceding neutral response, *t*(35) = 1.11, *p* = .277, *d*_*z*_ = 0.18.

#### Binding and retrieval effects on RTs

Figure 6a shows that participants responded more slowly after a preceding commission error than after a preceding correct response, *F*(1, 35) = 33.42, *p* < .001, η_p_^2^ = .49. Significant binding and retrieval effects emerged in faster correct response repetitions to target repetitions than to target changes, *F*(1, 35) = 65.65, *p* < .001, η_p_^2^ = .65. The interaction of both factors was significant, *F*(1, 35) = 5.04, *p* = .031, η_p_^2^ = .13, indicating that binding and retrieval effects were larger after a correct response, *t*(35) = 8.75, *p* < .001, *d*_*z*_ = 1.46, than after a commission error, *t*(35) = 3.38, *p* = .002, *d*_*z*_ = 0.56.

#### Binding and retrieval effects on percentages of commission errors

Percentages of commission errors were not significantly different between both preceding responses, *F*(1, 35) = 3.57, *p* = .067, η_p_^2^ = .09. Lower percentages of commission errors emerged for correct response repetitions to target repetitions than to target changes, *F*(1, 35) = 160.93, *p* < .001, η_p_^2^ = . 82. The interaction of both factors was significant, *F*(1, 35) = 15.94, *p* < .001, η_p_^2^ = . 31, indicating that binding and retrieval effects were larger after a correct response, *t*(35) = 17.36, *p* < .001, *d*_*z*_ = 2.89, than after a commission error, *t*(35) = 4.67, *p* < .001, *d*_*z*_ = 0.78.

### Discussion

In Experiment 3, we introduced a setup that allowed for investigating how agents represent the erroneous response after committing an action slip. Participants provided correct responses after an error more quickly if this response matched the preceding error than if the response corresponded to the neutral response. This result strongly suggests that the erroneous response remains in an activated state and thus continues to be accessible for future action planning. This continued activation of the erroneous response might prepare the ground for binding this response (e.g., its tactile action effects; Friedrich et al., 2020; Pfister, 2019) to any additional response-contingent changes in the agent’s environment (Foerster et al., 2022a).

The analysis of the percentage of commission errors for two successive errors depending on their (correct) response relation did not produce evidence for or against increased activation of the erroneous response. The analysis of this measure did also not support the account of bindings between the intended correct and the executed erroneous response. We still think that it would be worthwhile to pursue this research question further in paradigms that allow for an assessment of the hypothesized binding effects in RTs (Moeller & Frings, 2019a, 2019b, 2021), which we consider as a more powerful measure in this context. The observation of binding and retrieval effects between the intended correct and the erroneous response in percentages of commission errors would imply that the repetition of a target would activate the appropriate response first, which in turn would lead to the retrieval of the bound response, which would then also be executed despite an existing activation of the appropriate response. Instead, RTs of a following response that either does or does not match the potentially retrieved response would allow for a more fine-grained evaluation of the retrieval of bound responses.

Experiment 3 further replicated effects of goal-based binding in RTs and percentages of commission errors as we have observed before in Experiment 1 and 2 of this study and in previous work (Foerster et al., 2022a). This effect was of substantial size after an error although it was smaller than after a correct response, which we discuss in more detail in the following.

## General discussion

Binding and retrieval in action control has been studied mainly for correct action episodes, and theoretical accounts supported this empirical strategy by proposing that binding would only occur for successful responses (Hommel, 2005). The major assumption of the success-based account was only recently put to test by assessing binding and retrieval for action slips (Foerster et al., 2022a; Foerster et al., 2021; Parmar et al., 2022). For stimulus–response binding, the available evidence clearly supports an alternative, goal-based account of binding. The human cognitive system thus binds features of the concurrent stimulation to features of the intended but not actually executed responses in the face of errors. Following initial demonstrations of such binding effects, the present study provides a critical specification of previous work by highlighting that error feedback does not modulate binding and retrieval for action slips and, crucially, by studying the fate of the unbound erroneous response after goal-based binding.

Concerning the role of feedback, binding and retrieval effects to relevant stimuli after errors have consistently been observed to be smaller than corresponding effects after correct responses. The present findings show three independent replications of this pattern and rule out explanations in terms of limited error awareness for this effect. Given the current observation that explicit feedback plays no role in this modulation, it appears likely that this modulation roots in binding of features that only come available through response execution (Foerster et al., 2022a). The execution of a response produces sensory feedback, and such sensory feedback might be represented as features in bindings (Friedrich et al., 2020; Pfister, 2019). Crucially, these features would not be directly available but could only be predicted for the not executed, intended correct response in case of an error. The presence of binding and retrieval effects in the temporal feature RV_RT_ after a correct response but not after an error corroborates this interpretation further. Yet note that we found similar binding and retrieval effects across preceding responses in this measure before (Foerster et al., 2022a). Again, ΔRD as another measure of the temporal features of a response did not show any binding and retrieval effects, neither after a correct response nor after an error. An interesting avenue for future research will be the dissociation of binding for action slips of features that are closely connected to the execution of a response (such as the location of a to-be pressed key; Foerster et al., 2022a) and more abstract features (such as the classification of a to-be categorized object; Horner & Henson, 2009; Pfeuffer et al., 2017; Pfeuffer et al., 2018). Relatedly, we recently reanalyzed a selected additional aspect of the current data and collected novel data to explore the role of abstract features in terms of error-induced control states for binding and retrieval processes (Foerster, et al., 2022b).

The results of Experiment 1 and 2 further suggested that the erroneous response might continue to stay in an activated state after goal-based binding. Experiment 3 reinforced this notion by demonstrating that erroneous responses remain highly accessible in an upcoming action sequence relative to neutral control responses, while it did not show any evidence for binding and retrieval of the erroneous and the intended correct response. It thus seems as if erroneous responses remain highly accessible as an isolated representation unless it gets integrated with additional effects that follow sufficiently closely after action execution (Foerster et al., 2022a; Schwarz et al., 2018). This increased activation of the executed erroneous response might further hamper binding of the intended correct response to the stimulus by introducing response competition, resulting in smaller binding and retrieval effects than after a correct response where such conflict is absent. Still, the cognitive system succeeds in prioritizing the intended correct over the executed erroneous response in bindings to relevant stimuli. In other words, although the erroneous response might remain active, there will likely be a period in time where this response is not activated as strongly as the intended—and actually relevant—correct response. In line with that, findings from the visual attention literature point to a weaker activation of task-irrelevant than task-relevant features of the same object (O'Craven et al., 1999; Scolari et al., 2014). Further, empirical evidence showed weaker stimulus–response bindings for irrelevant than relevant stimulus features and for irrelevant than relevant response features, with no bindings between irrelevant stimulus and irrelevant response features (e.g., Pfister et al., 2022). These considerations allow us to further speculate why bindings between *relevant* stimuli and intended correct responses are smaller after an erroneous than after a correct response but similar-sized binding and retrieval effects emerge for *irrelevant* stimuli and intended correct responses regardless of response accuracy (Foerster et al., 2021). As both, stimulus and erroneous response features are irrelevant, they might not interfere with each other. Picking up the argument from the previous paragraph, only abstract but not execution-based features of the intended correct response might be subject to binding for irrelevant stimuli, and these abstract features should be equally available for correct and erroneous action episodes.

The available evidence thus points to a flexible and highly efficient architecture: Stimulus–response binding draws on features of an intended action in case of action slips, whereas it does not affect the erroneous response itself so that this response can enter bindings with response-contingent effects. Whether both bindings can be compiled in parallel or whether the cognitive system only compiles either stimulus–response or response–effect bindings in the wake of an action slip remains to be tested. A further intriguing question is whether goal-based binding after errors is an exhaustive description of binding also following correct responses—except for the higher number of available features in the latter case. Error commission commonly triggers thorough action evaluation, especially when errors arise during speeded responding (Jentzsch & Dudschig, 2009; Rabbitt & Rodgers, 1977; Scheffers & Coles, 2000; Steinhauser & Andersen, 2019; Welford, 1959). Deliberate action evaluation likely involves situation models of the environment, relevant task rules and action–effect contingencies (e.g., Wurm et al., 2020), and goal-based binding could be triggered through such internal simulations (Cochrane & Milliken, 2019). Similar operations can be assumed for those correct responses that are followed by a sufficiently strong evaluation, but binding may also take place on the fly for correct responses that do not receive any further processing.

The observation that binding for action slips tunes behavior toward future success ultimately begs the question of how lasting such binding and retrieval effects really are. Binding seems to be the ideal precursor of sustained learning against the background of these results. However, evidence to date suggests that bindings decay after several seconds (Frings, 2011; Hommel & Colzato, 2004; Hommel & Frings, 2020) and that binding and learning operate independently (Colzato et al., 2006; Moeller & Frings, 2017). Still, theoretical considerations on the compilation and access to short-term bindings and long-term instances of stimuli and responses are strikingly comparable (Logan, 1988, 1992). A common investigation of the role of errors for both domains might provide valuable insight into the interplay of integrated short-term and long-term representations of perception and action. A fruitful step in this direction could be the investigation of goal-based binding in guessing situations and how such effects relate to memory performance in the long run. Further, control adaptation is a hallmark of error processing (Crump & Logan, 2013; Jentzsch & Dudschig, 2009) and an open question is whether such adapted control states are subject to binding and retrieval as other control states (Dignath et al., 2019) as well as whether they could be stored in a sustained fashion as part of long-term instances (Logan, 2017).

### Conclusions

The current study consolidates theorizing on binding and retrieval in action control by showing that binding operates for successful and unsuccessful action episodes alike. In case of unsuccessful action episodes, the current findings reinforce the notion that episodic binding integrates the acted upon stimuli with the intended correct response. Moreover, the executed erroneous response remains in a state of high accessibility, likely functioning as preparation for binding of this response with potential (after)effects of an error. Binding and retrieval therefore appear adaptive for two reasons. First, for integrating stimuli and their correct responses, and second, for integrating erroneous responses and their contingent effects. These two faces of binding and retrieval pave the way for efficient responding in the future while also leveraging information about potential action-effect contingencies in the agent’s environment.

## Data Availability

The preregistrations (Experiments 1 and 2: osf.io/978ym, Experiment 3: osf.io/e65h7), experimental programs, data and the analyses syntax of all experiments are publicly available at the Open Science Framework (Experiment 1 and 2: osf.io/u837c, Experiment 3: osf.io/t25nb).

## Appendix

Appendix Tables 1, 2 and 3

**Table 1:** Descriptive data of Experiment 1

Preceding response	Condition sequence	RT in ms	Commissions in %	Omissions in %	RV_RT_ in ms	ΔRD in ms
Correct	Target repetition | correct response repetition	394 (21)	2.4 (1.9)	1.6 (1.3)	8 (3)	18 (5)
	Target change | correct response repetition	428 (24)	10.7 (4.5)	5.3 (3.4)	11 (3)	19 (5)
	Target change | correct response change	456 (30)	16.6 (10.7)	7.6 (7.3)	8 (2)	26 (9)
Error	Target repetition | correct response repetition	427 (26)	8.9 (7.9)	5.1 (6.8)	14 (10)	37 (20)
	Target change | correct response repetition	441 (35)	8.5 (7.4)	7.1 (8.4)	14 (12)	38 (19)
	Target change | correct response change	454 (31)	12.1 (11.6)	10.6 (10.1)	9 (4)	29 (19)

Mean response times (RTs) in ms, commission and omission errors in %, relative variance of RTs (RV_RT_) in ms, and absolute differences in response duration (ΔRD) between successive trials in ms with respective standard deviations (*SD*) in brackets as a function of preceding response and condition sequence

**Table 2:** Descriptive data of Experiment 2

Preceding response	Condition sequence	RT in ms	Commissions in %	Omissions in %	RV_RT_ in ms	ΔRD in ms
Correct	Target repetition | correct response repetition	393 (26)	2.3 (2.0)	1.6 (1.6)	8 (6)	18 (5)
	Target change | correct response repetition	430 (28)	13.2 (8.2)	6.0 (6.0)	11 (7)	19 (5)
	Target change | correct response change	460 (27)	17.7 (12.6)	7.3 (6.2)	7 (2)	26 (9)
Error	Target repetition | correct response repetition	432 (30)	10.4 (9.5)	7.9 (5.8)	11 (5)	35 (12)
	Target change | correct response repetition	445 (28)	10.9 (8.7)	8.3 (7.4)	11 (6)	35 (11)
	Target change | correct response change	460 (30)	13.0 (9.8)	11.0 (8.6)	9 (5)	27 (8)

Mean response times (RTs) in ms, commission and omission errors in %, relative variance of RTs (RV_RT_) in ms, and absolute differences in response duration (ΔRD) between successive trials in ms with respective standard deviations (*SD*) in brackets as a function of preceding response and condition sequence

**Table 3:** Descriptive data of Experiment 3

Preceding response	Condition sequence	RT in ms	Commissions in %
Correct	Target repetition | correct response repetition	517 (30)	8.5 (5.1)
	Target change | correct response repetition	549 (26)	27.5 (9.0)
Error	Target repetition | correct response repetition	550 (29)	16.3 (11.2)
	Target change | correct response repetition	567 (37)	25.8 (12.3)

Mean response times (RTs) in ms and commission errors in % with respective standard deviations (*SD*) in brackets as a function of preceding response and condition sequence
